# New therapeutic approach to Tourette Syndrome in children based on a randomized placebo-controlled double-blind phase IV study of the effectiveness and safety of magnesium and vitamin B6

**DOI:** 10.1186/1745-6215-10-16

**Published:** 2009-03-10

**Authors:** Rafael Garcia-Lopez, Emilio Perea-Milla, Cesar Ruiz Garcia, Francisco Rivas-Ruiz, Julio Romero-Gonzalez, Jose L Moreno, Vicente Faus, Guadalupe del Castillo Aguas, Juan C Ramos Diaz

**Affiliations:** 1 Department of Anaesthesia and Reanimation, Hospital Costa del Sol, Ctra Nacional 340, km 187, 29603 Marbella, Spain; 2 Research Support Unit, Hospital Costa del Sol, Ctra Nacional 340, km 187, 29603 Marbella, Spain; 3 CIBER Epidemiología y Salud Pública (CIBERESP), Spain; 4 Department of Paediatrics, Hospital Costa del Sol, Ctra Nacional 340, km 187, 29603 Marbella, Spain; 5 Department of Paediatrics, Children's and Maternal Hospital in Granada, Av. Fuerzas Armadas n° 2, 18014 Granada, Spain; 6 Department of Pharmacy, Hospital Costa del Sol, Ctra Nacional 340, km 187, 29603 Marbella, Spain; 7 CS La Carihuela, Torremolinos, Spain; 8 Hospital de Antequera, Av. Poeta Muñoz Rojas, 29200, Antequera, Spain

## Abstract

**Background:**

Tourette Syndrome (TS) is a neurological condition presenting chronic motor and phonic tics, and important degree of comorbidity. Considered an uncommon illness, it first becomes apparent during childhood. Current standard treatment only achieves partial control of the condition, and provokes frequent, and sometimes severe, side effects.

**Methods and design:**

Main aim:

To show that, with respect to placebo treatment, the combination of 0.5 mEq/Kg magnesium and 2 mg/Kg vitamin B_6 _reduces motor and phonic tics and incapacity in cases of exacerbated TS among children aged 7–14 years, as measured on the Yale Global Tic Severity Scale (YGTSS).

Secondary aims:

Assess the safety of the treatment.

Describe metabolic changes revealed by PET.

Measure the impact of the experimental treatment on family life.

**Methodology:**

Randomized, blinded clinical trials. Phase IV study (new proposal for treatment with magnesium and vitamin B_6_). Scope: children in the geographic area of the study group. Recruitment of subjects: to include patients diagnosed with TS, in accordance with DSM-IV criteria (307.23), during a period of exacerbation, and provided none of the exclusion criteria are met. Instrumentation: clinical data and the YGTSS score will be obtained at the outset of a period of exacerbation (t0). The examinations will be made after 15 (t1), 30 (t2), 60 (t3) and 90 days (t4). PET will be performed at the t0 and t4. We evaluated decrease in the overall score (t0, t1, t2, t3, t4), PET variations, and impact made by the treatment on the patient's life (Psychological General Well-Being Index).

**Discussion:**

Few clinical trials have been carried out on children with TS, but they are necessary, as current treatment possibilities are insufficient and often provoke side effects. The difficulty of dealing with an uncommon illness makes designing such a study all the more complicated. The present study seeks to overcome possible methodological problems by implementing a prior, phase II study, in order to calculate the relevant statistical parameters and to determine the safety of the proposed treatment. Providing a collateral treatment with magnesium and vitamin B_6 _could improve control of the illness and help reduce side effects.

This protocol was approved by the Andalusian Government Committee for Clinical Trials (Spain).

This study was funded by the Health Department of the Andalusian Regional Government and by the Healthcare Research Fund of the Carlos III Healthcare Institute (Spanish Ministry of Health).

**Trial Registration:**

Current Controlled Trials ISRCTN41082378

## Background

Tourette Syndrome (TS) is a condition that was first described in 1885 by Gilles de la Tourette as a clinical situation with significant tics, coprolalia and inappropriate behaviour. For several decades, it was considered a psychiatric disorder. This situation changed in the 1960s with advances in our understanding of neuroleptic drugs that made it possible to achieve some improvement in the symptoms presented by TS, now viewed as a neurological disorder. This observation constituted the beginning of the search for neurobiological factors for TS [1].

TS is currently considered a neurological condition with a genetic background and varying degrees of penetration, presenting chronic motor and phonic tics, sometimes accompanied by a comorbid pathology, and with a natural rhythm of remissions and exacerbations [2,3].

Diagnosis of TS is a clinical decision; it is defined as the presence of motor and phonic tics with an evolution exceeding one year, appearing during infancy or childhood (always before the age of 18 years) and often accompanied by a comorbid neurological disorder. This accompanying pathology is not always present, but normally determines the severity of the complaint. It consists of the existence of an attention deficit/hyperactivity disorder (ADHD), an obsessive/compulsive disorder (OCD) or other behavioural disorders such as poor control of impulses or a tendency to self-harm [2].

Tics are believed to be the most frequently occurring neurological disorder during childhood (up to 4%). Some authors have calculated the prevalence of TS to be 4–5 cases per 10,000 persons, counting all ages, but it is more frequent among children, affecting almost 1% of the juvenile population [4-7]. It predominantly affects males (75%). There is a clear tendency for various members of the same family to be affected, with varying degrees of severity, and it has been related with many chromosome sites, including 11q23. Symptoms among children are more intense when the transmission is bilinear (maternal and paternal) [2,8,9].

Tics can be motor and/or phonic, and may not initially be recognized as such. They begin as simple motor tics, frequently expressed by blinking or sniffing, while complex motor tics take the form of coordinated movements of the neck or unusual facial expressions. The presence of phonic tics is a diagnostic criterion; sometimes, these are simple, such as the emission of guttural sounds, while less frequently they take complex forms like echolalia or coprolalia, which are the most striking manifestations of the disorder. Although rare, the latter can have very negative consequences for the social and emotional life of the child.

Tics first appear at around the age of seven, beginning as simple ones and subsequently becoming more complex. By definition, they are long-lasting (more than one year's duration). They tend to worsen and improve over periods of two to three months. New tics may appear and others disappear, or reappear later. In general, tics evolve from simple to more complex forms, and from the head to the lower limbs. Great distress is caused to the child, who tries to suppress them, and may succeed for a limited time, after which they may suddenly reappear, stronger than ever. When tics are severe, they interfere with the life of the child [1,10].

The natural evolution of TS generally begins with ADHD at an early age (three or four years), with the first tics as such appearing at seven or eight years, first in the form of simple motor tics affecting the face, and then complex, intense and verbal. Subsequently, OCD may appear. The condition worsens at the age of ten to fourteen years, and usually improves by sixteen to twenty years. Fifty per cent of such patients are free of tics by the age of eighteen years, while the remainder continue to present the symptoms during adult life, although generally in a less intense, more manageable form that enables them to adjust and enjoy a normal social, family and emotional life [2]. Typically, the condition worsens in situations of stress or when catecolaminergic or corticoid medication is taken.

The pathogenesis of the complaint is not well understood, although family incidence is evident. The natural evolution during puberty, together with the greater incidence of tics among boys, are suggestive of a hormonal influence. One factor suggested is that of a synaptic neurotransmitter disorder resulting from the disinhibition of the cortical striatal thalamic circuit, on the basis of the relative degree of control achieved with neuroleptic medication [11-13]. Other possibilities suggested by the comorbid symptomatology (ADHD and OCD) are the basal ganglia (caudate nucleus) and the lower prefrontal cortex. However, postmortem examinations have failed to reveal specific alterations. No models of TS have been observed among animals. Neuroimaging studies are usually normal, although the presence of tics has been related to the presence of asymmetry in the basal ganglia [14]. It has been suggested that a disorder of the central neurotransmitters may give rise to TS, as a response to the modulation of the dopaminergic system; also mentioned as possible causes have been low levels of serotonin, glutamate and AMP; on occasion, too, PET has revealed an increased density of transported presynaptic dopamine and of D2 postsynaptic dopamine receptors [15-17]. Autoimmune phenomena have also been observed in this respect, with the presence of antineuronal antibodies against beta-haemolytic streptococci, as the case in other neurologic disorders (such as Sydenham chorea). It is in this context that the PANDAS syndrome (*Paediatric autoimmune neuropsychiatric disorders associated with streptococcal infections*) has been reported, although in the latter case, the appearance of tics is much more brusque [18]. The results of complementary studies of TS children are usually normal.

Treatment of TS is symptomatic. The tics are treated with neuroleptic blockers of dopamine receptors (D2), such as haloperidol. In recent years, other atypical neuroleptic drugs (pimozide, risperidone) have been incorporated into the treatment; these present fewer side effects, although they are not totally free of adverse reactions [19]. Some side effects, such as tardive dyskinesia may be severe and difficult to treat [20]. Clonidine (often with methylphenidate) is commonly used to treat Tourette's syndrome [1,2]. These drugs have been applied both singly and in combination, and in short term treatments (coinciding with periods of exacerbation) and long term ones [21-23].

ADHD is treated with amphetamines (methylphenidate) [24]. OCD is alleviated with the administration of antidepressives to inhibit serotonin reuptake (fluoxetine) [25,26].

In addition to pharmacological treatment, it is very important to provide suitable educational counselling for the child and his/her family. Cognitive-behavioural therapy can achieve an improvement in the comorbid pathology, especially as regards the obsessive-compulsive disorder [10].

### Background to the study

Other than in scientific texts, there exist anecdotal communications of the experiences of parents of TS children, reporting a significant decrease in the number and severity of tics suffered when certain multivitamin and mineral compounds were taken. This improvement, apparently, was notable but not total, and the symptoms generally reappeared when these substances ceased to be administered. This kind of communication constitutes a new physiopathological viewpoint, according to which a magnesium insufficiency might be the principal causative factor and the common pathway leading to the symptoms of TS and its comorbid pathology [27].

We wish to study whether any benefit is gained from the oral administration of magnesium and vitamin B_6 _to TS children. Both substances are commonly included in vitamin supplements for children and adults, they are considered very safe and may be acquired without a medical prescription.

Magnesium pidolate is taken as a syrup, at home. It is contraindicated for persons with illnesses such as decompensated or acute renal insufficiency, myasthenia gravis, diabetes or Cushing's disease. It should be utilized with care for patients with irregular calcium metabolism. The excipient may provoke allergic reactions, including asthma, especially among persons allergic to acetylsalicylic acid. No side effects have been described for therapeutic dosages. It should not be administered in conjunction with calcium (dairy products), which is prejudicial to its absorption.

Pyridoxine alpha-ketoglutarate could also be taken as a syrup, at home. No contraindications or side effects have been described, for therapeutic or even greater dosages. Its excipient, too, may give rise to allergic reactions, including anaphylaxia and bronchial spasms, especially among patients with a previous history of allergy or of asthma. It should not be mixed with other medicines, in order to preserve its absorption capacity.

We have designed a clinical trials to evaluate the effects of magnesium and vitamin B_6 _supplements on the clinical condition of children with TS. This supplementation is authorized for children and already commercially available; therefore, our aim in the present study is to confirm a possible new indication. The study should include children up to the age of 14 years, diagnosed with TS; this age limit was chosen as it is then when a standstill or remission of the symptoms may occur. The minimum duration of treatment and follow-up for these children should be three months, in view of the natural evolution of the illness, with spontaneous remissions and exacerbations. The symptoms most easily measured are the presence and severity of tics.

We present a new therapeutic approach to TS, based on the hypothesis that a deficit of vitamins or oligoelements, or a reduction of their activity, aggravates this pathology and often provokes severe comorbidity. Moreover, we take into account that current treatment protocols are frequently ineffective among many children and present important potential side effects.

Thus, we propose the following:

Main goal – to show that, with respect to placebo treatment, the combination of 0.5 mEq/Kg magnesium and 2 mg/Kg vitamin B_6 _reduces motor and phonic tics and incapacity in cases of exacerbated TS among children aged 7–14 years.

Secondary goals – to assess the safety of the treatment, in terms of adverse events; to describe metabolic changes resulting from the treatment, as revealed by PET; and to measure the impact of this treatment on the quality of family life.

## Methods and design

### Design

#### Type of clinical trials

Blind, randomized clinical study, Phase IV (new indication for supplementary magnesium and vitamin B_6_).

This study began in October 2007 and is currently in progress.

#### Recruitment of patients

**Figure 1:**
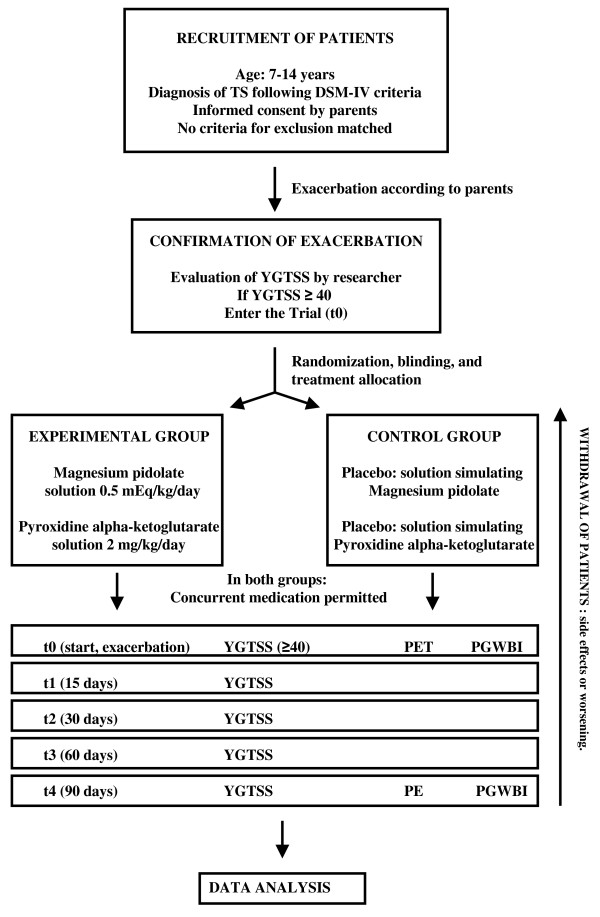
**Patients flowchart**.

The patients to be included are those diagnosed with TS, according to DSM-IV (307.23) criteria [28], currently in a phase of exacerbation. The primary healthcare paediatricians in the whole region were informed about the EECC, so that patients could be referred to the clinics where the study was being carried out. The doctors responsible for making the scale evaluation when a patient was included in the study referred him/her to the pharmacy department to be included in one of the two study groups, using the randomization table. Informed consent must be obtained from parents or guardians, and none of the exclusion criteria must be present. (See Figure 1).

### Study subjects

#### Patients

Patients diagnosed with TS, aged 7–14 years, either sex, with a minimum score of 40 points on the *Yale Global Tic Severity Scale (YGTSS) *[29], among whom the onset of clinical exacerbation has been observed.

### Selection criteria

#### Criteria for inclusion

- Aged 7 – 14 years, either sex. This is the age bracket during which the natural course of the illness is most exacerbated. Before the age of 7 years, the tics may not yet have appeared (this generally occurs at the age of 5 – 7 years). After 14 years, symptoms tend to stabilise.

- Informed consent of the child's parents or guardians, and reasoned agreement with the child.

- Clinical diagnosis of TS, according to Diagnostic and Statistical Manual of Mental Disorders – Fourth Edition (DSM-IV) criteria.

- Score of 40 or more on the YGTSS.

#### Criteria for exclusion

- Severe ADHD or OCD, not clinically controlled.

- Severe TDAH or TOC, not clinically controlled.

- Autism.

- Unrelated depression.

- Allergy to acetylsalicylic acid (due to the excipients used).

#### Randomization criteria

- Criteria set out above (age, diagnosis, consent).

- Evidence of exacerbation (Score ≥40 on the YGTSS).

- No contraindication due to the exclusion criteria.

- Patients who fulfil these criteria will be included, randomly, in one of the groups.

#### Randomization, blinding and assignment to treatment group

Randomization will be centralized and performed immediately after the inclusion of an eligible patient. The allocation envelopes will be serially numbered to ensure that allocation concealment is maintained within the pharmacy department at each hospital. A set of opaque envelopes marked with the letter A or B will be used to include patients in either the experimental or the control group. These envelopes will be kept in the pharmaceutical department responsible for dispensing the corresponding medication. Randomization to either the treatment or the placebo group will only be performed when a patient, suffering exacerbated TS, is considered eligible to receive the medication included in this study.

#### Instrumentation

The clinical data and the YGTSS score at the onset of the period of exacerbation of the clinical condition (t0) will be noted. The parents/guardians will be informed, and on receipt of their informed consent, the previously randomized medication will be provided. This medication is to be taken at the patient's home, and follow-up will be performed, at the healthcare clinic, at 15(t1), 30(t2), 60(t3) and 90(t4) days. PET will be performed at the start and end of the experimental period, for 15 patients. The psychological impact of the treatment on the families concerned will be measured using the Psychological General Well-Being Index (PGWBI) [30].

#### Evaluations

The clinical diagnosis of TS will be confirmed, and the YGTSS score ascertained, so that the patient may be included in the study and any subsequent fall in the global score recorded (at t0, t1, t2, t3 and t4). Metabolic changes in baseline and post-treatment PET will be recorded. The PGWBI will be determined at the start and end of the study period.

#### Withdrawal of individual patients

Patients may withdraw from the study at any time, for any reason and without suffering any sanction for doing so. The researcher-collaborator, after consulting with the principal investigator and the study coordinator, may also interrupt the treatment programme if the fact of continuing this treatment, in his/her opinion, would be prejudicial to the patient's welfare. If a patient withdraws or is withdrawn from the study, follow-up to day 90 should be continued whenever possible.

#### Follow-up of patients who withdraw from the study

Any patients whose treatment is withdrawn will continue to be followed up until the event in question is resolved or until, in the researcher's opinion, important changes in the patient's health state are unlikely to occur. The follow-up reports at 15, 30, 60 and 90 days will be completed for all patients who received medication (including placebo) during this study.

#### Suspension of the study

Should there occur severe adverse events related to the administration of the treatment, the study will be interrupted, and the researchers and the coordinator will decide whether to continue or not. Ultimate responsibility for this decision will rest with the coordinator. The relevant clinical research ethics committees and the healthcare authorities shall be informed of any decisions taken to interrupt, abandon or continue the study.

### Ethical criteria

#### Applicable regulations

The study will be carried out in accordance with the principles of the Helsinki Declaration, specifically the EMEA/CPMP declaration on the use of the placebo in clinical trials, with respect to the revised Helsinki Declaration, and in accordance with the guideline for Good Clinical Practice (CPMP/ICH/135/95 – 17 July 1996), as well as local regulations.

#### Recruitment

The study protocol was submitted to the Ethics Committee of the Hospital Costa del Sol (Marbella, Spain) for approval. Implementation of the study began after the Spanish national healthcare authorities formally approved it. Although patients will be informed that they are free to abandon the study at any time, we shall seek to recruit those offering the maximum probability of remaining within the study until its conclusion.

#### Informed consent for minors

After identifying candidate patients for inclusion in the clinical trial, the children will be given an oral and written explanation of the study. The parents/guardians will be provided with all available information and any complementary information they might require, and they will be given an information sheet so that their informed consent for the child to participate in the trial may be obtained. Once this has been signed, the form should be given to the researcher when the child attends the clinic for treatment of exacerbated TS (t0).

#### Liability for injury

An insurance policy for civil liability will be subscribed to cover any injuries that may arise from the performance of the study.

### Treatment details

#### Dosage and administration of medication

The medication used in the trial will be administered orally, at the patients' homes. The following medication will be provided:

Magnesium pidolate 0.5 mEq/Kg/day, divided to be taken twice daily. This should not be taken in conjunction with calcium or dairy products.

Pyroxidine alpha-aketoglutarate 2 mg/Kg/day, once daily.

#### Preparation and labelling of treatment procedures

The medication for the trials will be prepared, labelled and stored by the pharmaceutical service. The active principles of the treatment group were obtained via commercially available drugs. The placebo used was created in the hospital's pharmacy department, emulating the excipients and volume of the experimental medication. Procedures for reducing the volume of medication per pack will be implemented in accordance with ICH requirements. The study coordinator will supervise all procedures applied in this respect.

#### Other medication allowed

The patients will continue taking their usual medication to control TS symptoms or associated comorbid pathologies. Moreover, they will continue receiving any pre-existing psychological support treatment.

In addition, they will continue taking any medication prescribed prior to their entering the study, for any other concurrent illness.

### Specific methods

#### Evaluation of effectiveness

The clinical evaluation of the patients' tics will be carried out by applying the Yale Global Tic Severity Scale (YGTSS) [29]. This scale has been validated for the study of motor and phonic tics for children with TS, in comparison with other scales such as the Shapiro Tourette Syndrome Severity Scale and the Tourette Syndrome Global Scale. It was designed for studying TS and other disorders that provoke tics, and functions by evaluating the number, frequency, intensity, complexity and degree of interference of motor and phonic tics. The YGTSS is applied by means of a semi-structured interview with multiple informants (generally, the parents) who assess the child's tics over a period of at least one week. The translation into Spanish and its adaptation to local conditions were previously validated by the authors of the present paper, in a prior study [31].

#### Measurement instruments

**The Yale Global Tic Severity Scale**. The Spanish version of the YGTSS will be used. Scoring on this scale ranges from 0 to 100 points. A score of 40 or more on the YGTSS at t0 is defined as a state of exacerbation and therefore a necessary condition for the patient's inclusion in the study.

**PET **will be carried out at the beginning and end of the study in order to detect metabolic changes that may be related to the effectiveness of the treatment. Qualitative changes in the metabolism pattern within the prefrontal cortex and in the basal nuclei will be evaluated. This will be complemented with a quantitative study of the normalized rates of metabolism in such zones.

The Spanish version of the **Psychological General Well-Being Index **will be calculated for the parents, at the beginning and end of the study in order to assess the psychological repercussions of the treatment on family life [30,32]. This scale indicates the subjective feelings and psychological well-being (or otherwise) during the past week.

#### Adverse events

Any adverse event notified spontaneously by the subject, or observed by the researcher or by the research team will be recorded on the form designed for this purpose. The researcher will classify the intensity of adverse events in accordance with the following scale:

- *Mild*: some discomfort experienced but not such as to interrupt normal daily activity.

- *Moderate*: sufficient discomfort to reduce or notably affect normal daily activity.

- *Severe*: provoking incapacity to work or perform normal daily activity.

The periodicity of the event will be classified in accordance with the following scale:

- *Single occurrence*: just one event, of limited duration.

- *Intermittent*: various episodes of an event, each one of limited duration.

- *Persistent, unlimited*: an event that has persisted over time, and is of indefinite duration.

For each adverse event, its relation with the medication taken (definitive, probable, possible, improbable, none), in the researcher's opinion, as well as any action taken as a result, will be recorded on the data collection form. The occurrence of an adverse event that is fatal, potentially fatal or incapacitating, or that requires or prolongs hospitalization, or that provokes severe congenital anomalies will be recorded as a "severe" adverse event (SAE).

All SAEs and unexpected adverse pharmacological reactions (UAPR), defined as adverse events whose nature or intensity is not in accordance with any adverse event expected, will be notified by the researcher to the study coordinator by phone, mail or fax as soon as is reasonably possible, but in any case within 24 hours of their occurrence.

#### Follow-up after occurrence of an adverse event

All adverse events will be observed until their remission or stabilization. Depending on the circumstances, this observation might necessitate evaluation by and/or referral to the patient's GP or to a specialist.

### Procedures and control

#### Selection of subjects

Patients diagnosed with TS will be included in a preliminary "potential subjects" group. Before undertaking any selection activity, written informed consent, signed and dated, must be obtained from the parents or guardians. The patients will be informed, before any action is taken, of the purposes and structuring of the study, and any doubts that might be expressed will be answered. It will be stressed that they have the unconditional right to withdraw from the study at any time. They will be asked to return to the healthcare clinic to begin the study procedure when they believe the child is entering a period of exacerbation of TS.

#### Study periods

t0 Entry into the study

   When the child's parents/guardians consider a period of exacerbation is beginning, they enter the active phase of the study. The YGTSS score is assessed, taking into account that a score of at least 40 is a prerequisite for entry into the study.

   The baseline PET is carried out for patients included in the study.

   The PGWBI for the parents/guardians is calculated.

**t1 At 15 days**.

   The YGTSS is calculated.

t2 At 30 days

   The YGTSS is calculated.

t3 At 60 days

   The YGTSS is calculated.

**t4 At 90 days. End of study period**.

   The YGTSS is calculated.

   PET control.

   PGWBI for parents/guardians.

### Data analysis

#### Calculation of the statistical power; establishing the sample size; safety

The sample size was established by means of a prior pilot scheme based on a phase II effectiveness trial, with 10 patients monitored over 3 months. There was found to be an improvement of 50% on the baseline YGTSS score (mean total number of tics 12.9 (SD = 11.20) in the experimental group vs. 26.7 (SD = 7.38) in the control group). For the present study, we calculated a mean score of total tics of 13 (SD = 10) after 3 months for the experimental group, and of 23 (SD = 10) for the control group, for a level of significance of 0.05 and a statistical power of 0.8, taking the least favourable case. On the basis of these data, 17 patients per group (34 in total) were needed. This sample size was then over-dimensioned to allow for a possible dropout rate of 10%, and so the minimum sample size was calculated to be n = 38 (19 patients per group).

#### General considerations

A descriptive statistical analysis will be performed, using statistics of central trend and dispersion for the quantitative variables, and frequency distributions for the categorical ones, carried out separately for the experimental and control groups. The baseline variables will be compared using these techniques. A flow diagram will be drawn to show the sequence from the initially eligible population to the one finally included in the study (refusals, dropouts, lost to follow-up, etc.), in accordance with the criteria of the CONSORT guidelines.

A comparison will be made of between-group differences between the initial and final measurements for the diverse elements of the Yale Scale, using unpaired nonparametric tests. For the final within-group comparison, paired nonparametric tests will be used non-parametric Repeat measures with adjustments for baseline imbalances and scores. The same analysis will be performed for the index of psychological well-being, measuring the repercussions of the treatment on family life.

The principal outcome variable (POV) will be taken as the change in the global score on the Yale Scale, adjusted for the baseline values. A simple linear regression model will be made for this POV, and the level of significance set at p < 0.05. Subsequently, a multiple linear regression model will be created, including the variable "Experimental/Control Group", and adjusting for any baseline variables that might be unbalanced. Statistical adjustment was performed by means of a multivariate model including the variable group (experimental and control) and concomitant treatment (Yes/No).

The safety of the treatment will be described in terms of the percentage of adverse events, and according to levels of assignation of responsibility.

## Discussion

TS is considered to be a rare neurological illness, although increasingly high rates of prevalence are being reported in current studies. The condition is seldom diagnosed, due to ignorance of its existence and characteristics [7]. Until very recently, TS was only recognized as such for the most severe cases, in which there was an important degree of functional limitation and very evident coprolalia. Although this situation is changing, TS is still considered an uncommon disease.

Few clinical studies have been made with children, because, in addition to the normal difficulties arising in this kind of study (with adults), legal considerations must be borne in mind, due to the logical necessity to protect minors. Nevertheless, such studies are clearly needed [33].

TS is a neurological illness, and its physiopathological and inherited alterations, as well as aggravating environmental factors, are increasingly well understood [10]. Effective treatment is provided by dopamine post-synaptic D2 receptor-inhibiting neuroleptic agents [34,35]. Other types of medication, such as anti-epileptic drugs, have also been applied, although the evidence of their effectiveness is less apparent [36,37]. However, the control of TS symptoms provided by the latter kind of treatment is only partial, and side effects such as sedation and dysphoria are common, as are others that are potentially very serious (tardive dyskinesia, arrhythmia or sudden death) [20,38,39]. In view of these considerations, many other forms of treatment have been studied, of varying nature and effectiveness; these include drugs that act on the central nervous system, botulinum toxin, acupuncture, plasmapheresis, conventional neurosurgery and, more recently, deep brain stimulation [40-45]. This diversity suggests that the illness is, as yet, poorly controlled, especially in the most severe cases.

The possibility of a complementary treatment with magnesium and vitamin B_6 _would represent an important improvement in controlling the illness, by reducing the need for neuroleptic drugs and other medication; it would also reduce the amount and severity of side effects.

The alternative therapy proposed in this paper is based on theoretical principles, but also on specific communications and on a prior study, on which the present protocol is based [27].

In clinical terms, magnesium deficiency is related to neuromuscular hyperexcitability, and may give rise to convulsions, chorea and athetoid movements. It has also been related to biochemical and genetic alterations that may provoke the symptoms evidenced by children with TS [46].

The enzyme kynureninase requires the presence of both magnesium and pyridoxal phosphate, and so in cases of hypomagnesemia there are high levels of kynurenines in the blood – which is the case with TS. Abnormally high levels of kynurenines provoke anxiety, an increased release of noradrenaline, locomotory hyperactivity, tics, increases in quinolinic acid, heightened sexual activity among females, reduced levels of serotonin and blocking of the GABA receptors. Therefore, this enzyme has been related to the presence of tics, anxiety and coprolalia-copropraxia [47].

A deficit of magnesium reduces the activity of vitamin B_6_, by inhibiting the activity of the alkaline phosphatase required to achieve its active form in the tissues, pyridoxal phosphate. A deficit of vitamin B_6 _activity has been related with raised levels of kynurenines, spasmodic movements, abnormal movements of the head, hyperirritability, increased sympathetic stimulation and heightened sensitivity to glucocorticoids. The symptoms worsen in situations of stress and with the administration of catecolamines and glucocorticoids [48].

Magnesium deficit increases NMDA receptor activity, producing greater neural excitability. In consequence, there is heightened anxiety and orofacial tardive dyskinesia, an increased release of dopamine, more defensive behaviour and greater modulation of serotonin receptors. Furthermore, other symptoms may be affected, such as migraine, which is more frequent among patients with TS [49].

This situation also raises the levels of substance P, and is associated with defensive behaviour, heightened response to stress and to allergenic phenomena.

It should be noted, however, that to date no clinical trials have been published corroborating this hypothesis [50]. In view of the fact that current treatment with neuroleptic medication only achieves partial effectiveness and may cause severe side effects, we believe there is reason to carry out clinical trials with the proposed, less aggressive, substances [38].

The composition of multivitamin and mineral compounds is very diverse, but from a theoretical standpoint, taking into account their pharmacological properties and following the hypothesis set out above, we consider that clinical trials should be focused firstly on the administration of magnesium and vitamin B_6_. Both substances present a wide therapeutic margin and have very few side effects; moreover, they are authorized for similar indications among children and have a long history of therapeutic use.

Magnesium ions are fundamentally intracellular or located in the bones, with only 1% being extracellular; in consequence, magnesium levels in plasma do not accurately reflect the content in the body. Traditionally, it has been used intravenously to treat severe nutritional deficit, as an antiarrhythmic agent and in cases of eclampsia-preeclampsia. Orally-administered magnesium has been used as a nutritional supplement. It can be given to children. It is contraindicated in cases of acute or chronic decompensated renal insufficiency, myasthenia gravis, diabetic coma and Cushing's disease. The recommended physiological dose is 0.2–0.5 mEq/kg/day [51].

Vitamin B_6 _(pyroxidine) is a hydrosoluble vitamin with a wide therapeutic margin. In clinical and pharmacological trials, it has been shown to have interesting properties, participating in oxidative deamination, transamination and decarboxylation; it also participates in the decarboxylation of glutamic acid to GABA, from DOPA to dopamine and from 5-hydroxytrytophan to seratonin. It presents anti-convulsant properties and seems to exercise a neuroprotective and antitoxic effect. It can be administered to children, and has been authorized for use to treat children with alterations in character, language and behaviour; learning difficulties; delayed learning to walk; convulsive illnesses; intoxication of the central nervous system; trembling; and Parkinson's disease. The dosage provided may vary widely, as renal elimination ensures its toxicity is minimal [52].

As the activity of magnesium is a consequence of its ion fraction, it is mainly intracellular and bears little correlation with the magnesium in serum. We do not believe clinical conclusions can be drawn from the measuring of magnesium levels in serum [53]. In the same way, determining the levels of vitamin B_6 _in serum would not be useful, in view of the fact that the problem lies in the activity deficit [48].

The statistical parameters to be used in this protocol are derived from the above-mentioned prior study carried out by our research group; this was a phase II clinical trials of the effectiveness and safety of the treatment in question. It was useful for pre-determining the degree of improvement that could be expected among the experimental group and, therefore, the minimum sample size needed, together with the mean and standard deviation values measured on the YGTSS.

This prior study showed that the treatment produced a significant decrease in the YGTSS score, with no side effects. Nevertheless, this was a pilot study, with methodological limitations that we seek to overcome in the present protocol [54].

In turn, the methodological instrument that will be used was also developed in a previous study of ours, namely the YGTSS translated into Spanish, adapted and validated for the local population [31].

The population chosen for this study is deliberately restricted, being limited to children aged 7–14 years, which is the age group in which clinical exacerbation, in theory, is greatest. By this limitation, we seek to obtain a population group of very homogeneous characteristics and, at the same time, one presenting severe symptoms, defined as a high score on the YGTSS (≥40).

It may be difficult to recruit the necessary numbers of patients for the sample size envisaged, given the nature of the clinical condition, the relative rarity of its diagnosis and the fact that children are involved. We hope to overcome this difficulty by expanding the geographic scope of the study and the number of medical personnel involved in recruiting suitable patients.

The follow-up period of three months is based on the natural course of the illness, with exacerbations and remissions lasting approximately this length of time. We believe that if the patient enters the study at the onset of a period of exacerbation, the YGTSS score will be high and it will be easier to identify significant differences, with the symptoms being controlled to a greater extent, and more quickly, among the experimental group than among the control group.

The reason for performing a PET on the experimental group before and after the administration of the medication is to objectify the dopaminergic activity within the basal nuclei and in the prefrontal cortex, as well as to reveal any alterations occurring in these areas as a result of the medication administered. However, these images are of low specificity and might not enable clear-cut conclusions to be drawn. Nevertheless, it seems reasonable to attempt to support the results of the clinical evaluation with these alternative, objective data. Be that as it may, these images will be of use as a basis for subsequent studies [15,16].

It is also important to assess the repercussions on family life (which tends to be greatly impaired in severe cases) of an improvement in the control of TS symptoms among children. The PGWBI reflects psychological well-being (or otherwise); it is based on theories of evaluation of the domestic environment and is an appropriate means of determining the distortion produced by TS within the household [30,32]. We have no doubt that a direct correlation will be found between the children's symptoms and the psychological well-being within the family home.

The joint application of these three measurement methods, namely the objectification of tics and incapacity, the assessment of metabolic changes in the circuits of the basal ganglia and of the cortex, and the evaluation of stress within the family, will enable us to reach an objective judgement as to the effectiveness of the treatment being tested.

In summary, treatment for TS continues to present important shortcomings and further clinical trials are necessary in this respect, especially among children.

## Abbreviations

ADHD: Attention deficit/hyperactivity disorder; CONSORT: Consolidated Standards of Reporting Trials; DSM-IV: Diagnostic and Statistical Manual of Mental Disorders; GABA: Gamma aminobutyric acid; MPA: Monophosphate adenosine; NMDA: N-methyl-D-aspartate; OCD: Obsessive compulsive disorder; PANDAS: Paediatric autoimmune neuropsychiatric disorder associated with streptococcal infection; PGWBI: Psychological General Well-Being Index; POV: Principal outcome variable; SAE: Severe adverse event; YGTSS: Yale Global Tic Severity Scale

## Competing interests

The authors declare that they have no competing interests.

## Authors' contributions

RGL, JRG Clinical review of the subject and previous consideration. RGL, JRG, EPM, CRG, FRR Development of the phase II prior study. EPM, FRR, RGL Methodological design. CRG, RGL Preparation of documentation. CRG Preparation of the International Registry entry of the clinical trial. RGL, CRG, JRG Training with and standardization of procedures and clinical measurement instrumentation (YGTSS). CRG Review and design of the PET evaluation. JLM, VF, RGL Review and decision making standpoint on medication.
